# Scientific discrepancies in European regulatory proposals on endocrine disruptors—REACH regulation *quo vadis?*

**DOI:** 10.1007/s00204-021-03152-7

**Published:** 2021-09-10

**Authors:** Andreas Natsch

**Affiliations:** Fragrances S&T, Ingredients Research, Givaudan Schweiz AG, Kemptpark 50, CH-8310 Kemptthal, Switzerland

**Keywords:** Endocrine disruptor, Non-monotonic dose response, Reproducibility, No-threshold assumption, Regulation

## Abstract

The EU chemical strategy for sustainability places a high focus on endocrine-disrupting chemicals (ED), the importance of their identification with increased testing and a ban in consumer products by a generic approach. It is assumed that for ED no threshold and hence no safe dose exists, leading to this generic approach. This view appears to be linked to the claim that for ED ‘low-dose non-monotonic dose response’ (low-dose NMDR) effects are observed. Without this hypothesis, there are no scientific reasons why classical risk assessment cannot be applied to the ED mode-of-action. Thus, whether for ED low-dose NMDR effects are considered a reproducible scientific fact by European authorities is Gretchen’s question in this politicized field. Recent documents by the SCCS, EFSA and ECHA reviewed herein illustrate the diverging views within European scientific bodies on this issue. Furthermore, ED researchers never replicated findings on low-dose NMDR in blinded inter-laboratory experiments and the CLARITY-BPA core studies could not find evidence for reproducible NMDR for BPA. ECHA proposes a battery of in vitro tests to test all chemicals for ED properties. However, these tests were never validated for relevance and their high positivity rate could lead to increased follow-up animal testing. Based on (i) lack of reproducibility data for low-dose NMDR, (ii) diverging views within European authorities on NMDR and (iii) lack of fully validated in vitro test methods it might be premature to fast-track the wide-ranging changes in the regulatory landscape proposed by the authorities ultimately leading to drastically increased animal testing.

**Supplementary Information:**

The online version contains supplementary material available at 10.1007/s00204-021-03152-7.

## Introduction

In October 2020, the European Union published the ‘Chemicals Strategy for Sustainability’ (Strategy) as part of Europe’s Green Deal (Commission 2020). The strategy places a high focus on endocrine-disrupting chemicals (ED), the importance of their identification with increased testing and the ban of their use in all consumer products based on their generic classification as Substances of Very High Concern (SVHC). This is based on the view that EDs are chemical of ‘special concern’ (Commission 2020). The Green Deal and the Strategy, aiming at reducing environmental pollution and identifying chemicals of special concern can only be welcomed. However, when it comes to identifying chemicals of concern regarding effects on the endocrine system, some basic scientific principles need to be maintained and scientific shortcuts in this politicized field could lead to wide-ranging effects especially for the European cosmetic industry, small and middle sized enterprises and, most importantly, animal welfare and the number of animal tests conducted. Thus, based on the Strategy, the European Union discusses a rapid implementation of new regulations on chemicals introducing (i) a testing requirement on at least seven in vitro endpoints for endocrine activity for all chemical registrations (Commission 2021b) (ii) a new classification scheme for so called ‘endocrine disruptors’ (ED) (Commission 2021a) and (iii) a ‘generic approach’ banning all EDs from consumer products considering them harmful chemicals ‘requiring special attention’ (Commission 2020) irrespective of dose and exposure considerations.

This latter proposal is intriguing and it apparently is based on the assumption that for ED (similar to directly acting genotoxicants) no threshold and hence no safe dose exist which leads to this generic, hazard-based approach. This view on the lack of a toxicological threshold comes from the claim of a group of researchers that for most ED ‘low-dose non-monotonic dose response’ (low-dose NMDR) effects are observed as reviewed by these researchers in two detailed reviews (Vandenberg et al. 2012, 2013). Bisphenol A (BPA) is the chemical studied in most detail, and low-dose NMDR have been studied in most detail for the estrogenic mode-of-action of BPA. The consistency and reproducibility of the low-dose NMDR hypothesis has been challenged by a number of researchers including a leading group of toxicological experts (Autrup et al. 2020; Kamrin 2007; Rhomberg and Goodman 2012) and it has been scrutinized by the CLARITY-BPA study by the US National Toxicology program (Badding et al. 2019; Camacho et al. 2019; Dere et al. 2018). Reproducibility is a key ‘first principle’ in science and hence the question whether reproducibility was tested in a proper way is the first question for the assessment of low-dose NMDR effects. In the context of the proposed new European regulations, the key question is: do European authorities have a consolidated conclusion, whether the “low-dose NMDR” for ED indeed do exist and are considered an established, reproducible scientific fact? If this is not the case, there is no clear reason why for an ED mode-of-action (MoA) different regulatory tools are needed as compared to those applied already for similar reproductive and developmental effects triggered by a different MoA. This is the crucial, central question, as the whole discussion on ED is completely changed in case the low-dose NMDR claim cannot be substantiated. The position of European regulatory bodies on this question will be explored here based on some recent reports published over the last months: The evaluation and the ban of butyl paraben by Denmark/ECHA (DK-EPA 2020), the risk assessment of propyl paraben by the Scientific Committee for Consumer Safety (SCCS) (SCCS 2021) and the view on NMDR of BPA by the European Food Safety agency (EFSA) (EFSA 2021).

In case the assumptions on low-dose effects would be considered an established fact by the authorities, the question then is whether appropriate tools are available to study those effects in a regulatory setting. As will be reviewed here, the in vitro assays proposed to be applied to all chemical registrations in a new test battery (Commission 2021b) have been validated for reproducibility but not for relevance. Finally, a strong call to bring the toxicological principle of dose-dependence back into the assessment of ED by the leading toxicologists and editors of the key toxicological journals appears to be unheard (Autrup et al. 2020). Bringing these diverging views together in this commentary may help to realize to what extend the scientific questions have not been resolved with large scientific discrepancies being ignored in a rapid rush to action.

## Potency and metabolism: founding pillars of toxicology ignored in the ban of butyl paraben by ECHA

In fall 2020, the EU decided to label butyl paraben (BP) as an ED and hence a SVHC to be banned in Europe based on an Annex XV report (DK-EPA 2020). Use of BP had already before been abandoned by industry in Europe, thus there are no vested interests in this substance. Hence we can now look at the assessment report leading to its ban without economical interest in the substance in question to understand which evidence is currently considered as sufficient for the ban of a chemical as ED in the EU and how in vitro data, in vivo data and potency considerations are weighted to come to this conclusion.

The report states that in vitro there is sufficient evidence for estrogenicity of BP and that *“Several studies show an estrogen-receptor agonistic response [for BP] similar to estrogen”*. However, based on nine experiments in the Tox21 database (Huang et al. 2014), the AC50 of BP is at 1.3 × 10^–5^ M, while the value for 17β-estradiol (E_2_) is 5.6 × 10^–12^ M (OECD 2016). A potency difference of 6 orders of magnitude is also reported in the study by Pop et al*.* (Pop et al. 2018)*.* This potency difference of ≥ six orders of magnitude is not mentioned at all in the report, BP being claimed to have an *“agonistic response similar to estrogen”*. Furthermore, in seven instances, the in vitro studies are summarized in Annex III of the report with the blunt statement “*effects similar to estradiol (positive control)”*, without any indication of potency difference between BP and estradiol.

Parabens are rapidly metabolized in in vitro (Abbas et al. 2010) and in vivo (Aubert et al. 2012), and Aubert et al*.* reported that for butyl- and propylparaben all administration routes led to a single peak in plasma, corresponding to *para*-hydroxybenzoic acid. This detailed study is ignored in the Annex XV report, only four lines in the 149 page report are dedicated to toxicokinetics: “Butylparaben is metabolized to para-hydroxybenzoic acid (PHBA) and a large proportion of PHBA is excreted as p-hydroxyhippuric acid (PHHA, the glycine conjugate of PHBA). Recent studies indicate the presence of other metabolites in human urine. This [Annex XV] report does not include a review of metabolism.” The fact that estrogen-receptor binding (even of the very low potency observed) is only plausible for the parent and not the key metabolite and that metabolism is thus a key consideration in the MoA analysis is not mentioned.

The WHO definition for an ED requires that “it alters function(s) of the endocrine system and *consequently* causes adverse health effects in an intact organism”. Thus the adverse effect in the intact organism must be proven and a causal link between the endocrine effects and the adverse effects must be established. Whether an exogenous chemical at a given dose can have effects on a hormone receptor will depend on its concentration and binding affinity relative to the endogenous ligand and hence relative receptor occupancy by the external and internal ligands following classical Michaelis–Menten laws. Thus to establish plausibility for the link between the mechanistic in vitro data and in vivo effects, potency data and systemic availability of the exogenous ligand (and hence toxicokinetics) are essential and cannot be ignored. However, in the mode-of-action analysis and in the assessment of biological plausability there is a complete lack of considerations whether the low potency could make an estrogenic MoA biologically plausible at all.

The authors of the report might counter this criticism claiming that the Annex XV report is about hazard identification only and identifying BP as ED—and that hence metabolism and potency are of no relevance. However, this argument is scientifically invalid: internal dose and potential estrogen-receptor occupancy in the in vivo situation is critical to scientifically determine whether the estrogenic MoA is biologically plausible, even for hazard assessment. This can only be properly assessed by accounting for potency and toxicokinetics. Thus this report ignores all potency considerations for in vitro data in the MoA analysis and it ignores metabolism for such a rapidly metabolized molecule—intentionally omitting such key available data on these two founding pillars of toxicology from a comprehensive report seems not an appropriate way of assessing whether a chemical fulfills the WHO criteria for an ED.

## Toxicological principles rescued—SCCS assessment of propyl paraben

Interestingly, in the mandate to evaluate potential ED used in cosmetics, SCCS had to evaluate propyl paraben (PP) and this report appeared briefly after the BP ban (SCCS 2021). This report differs by 180° from the report on BP: Thus SCCS considered both potency and metabolism as key drivers. Table 5 in the SCCS report lists all in vitro studies, presents potency of both PP and E_2_ and highlights this potency difference in bold font concluding that: “These literature reports demonstrate that the estrogenic activity of PP is extremely weak (approximately 20,000–700,000-fold lower at maximum concentrations) compared to 17β-estradiol (references). When potency is so weak, it raises questions on the biological relevance of these findings.”

In regards to metabolism, the report highlighted the rapid cleavage of this ester and concluded “The available information from a number of ex-vivo, in vivo, and human studies indicates that propylparaben is rapidly absorbed following oral ingestion, metabolized, and eliminated through unrinary route (terminal half-life: 2.9 h). This indicates that accumulation of propylparaben in the body may not be of a concern for consumer safety.” This report also cited the seminal studies on metabolism of parabens of different side-chain lengths by Aubert et al*.* (Aubert et al. 2012) excluded from the report on BP.

Furthermore, SCCS highlighted that metabolism cannot be ignored when evaluating mechanistic in vitro data stating that: “However, it remains difficult to extrapolate data from in vitro assays to humans. Due to a rapid metabolism of parabens in vivo, it is unlikely that estrogenic effects through direct estrogen/androgen receptor activation by parent parabens can cause harmful effects in humans.”

Finally, the SCCS report weighted guideline-compliant studies higher as compared to literature reports and also for in vivo studies reported potency differences vs. E2. They concluded that: “The SCCS is of the view that, although the available data on propylparaben provide some indications for potential endocrine effects, the current level of evidence is not sufficient to conclusively regard it as an endocrine-disrupting substance or to derive a specific endocrine-related toxicological point of departure for use in safety assessment.” SCCS progressed to a classical risk assessment to determine margin of safety. Based on this, SCCS consider PP safe for use in cosmetics as currently allowed.

Thus these two assessments on two very similar chemicals rapidly metabolized to the same circulating metabolite stand in a stark contrast to each other: the assessment of BP appears based on the idea that in vitro potency and metabolism is irrelevant for ED MoA assessment while the assessment of PP by SCCS carefully discussed in vitro potency and metabolism and their impact on identifying an ED and finally followed classical principles of risk assessment. The question then is: Which approach will EU authorities consider the correct approach for Toxicology in the 21st century and under the Chemicals Strategy? More importantly, we have to ask what leads to these completely divergent views. The unconventional view that potency of receptor binding should not be considered nor even mentioned can only be inspired by the low-dose NMDR assumption, stating that chemicals with very weak estrogenic potency may still have effects at exceedingly low concentrations in vivo*.* This leads to the crucial question to be answered first: is the low-dose NMDR assumption a reproducible scientific fact?

## Science’s first principle: replication—are low-dose NMDR effects replicated in independent repetitions?

The number of studies reporting low-dose NMDR effects is very large, and Vandenberg et al*.* have argued, that low-dose NMDR effects are thus no longer a hypothesis but an established fact as this can be assessed in a weight of evidence approach (Vandenberg et al. 2012). On the other hand multiple authors contested the reproducibility based on the wide range of different effects observed in different studies and absence of effects in other studies (Autrup et al. 2020; Kamrin 2007; Rhomberg and Goodman 2012). The plethora of studies on low-dose NMDR investigated a multitude of different endpoints in a multitude of different treatment regimens at widely diverging doses and using very different statistical approaches—but what is still lacking after 25 years of extensive research in this field is a multi-laboratory ring trial with blinded samples to prove that at least one of the key effects is observed at a similar dose, into the same direction and with an (adverse) effect of similar magnitude on a pre-defined endpoint when samples spiked with a putative ED (such as BPA) are tested in multiple laboratories in a blinded manner. Blinded, multi-laboratory trials have become the gold standard and in vitro toxicological methods are only accepted by the OECD or by ECHA and other regulatory bodies if they are properly validated with such studies. This is done even for relatively simple protocols following classical principles, for which reproducibility could even be anticipated without such scrutiny. However, in the field of low-dose NMDR for ED, which puts in question the central paradigms of toxicology and pharmacology, the new paradigm is maintained for 25 years and is starting to influence regulatory decision making despite the complete lack of multi-laboratory ring trials.

The lack of replication for low-dose NMDR observations has been highlighted repeatedly by different authors, specifically e.g. by Kamrin (Kamrin 2007). Interestingly the leading ED researchers have responded to Kamrin’s criticism in their key seminal review on low-dose NMDR in detail (Vandenberg et al. 2012), stating: “First, suggesting that reproducibility is equivalent to the same results obtained each time a study is conducted is unrealistic and not a true representation of what is required of replication. As has been discussed in other fields, “there is no end to the ways in which any two experiments can be counted as the same—or different... All experiments are the same in respect of their being experiments; they are all different by virtue of being done at different places, at different times, by different people, with different strains of rat, training regime, and so on “(73)”*.* This statement undermines science’s first principle: that an experiment should give the same result independently of whom does it, when and where. Of course in a multi-laboratory trial one would use the same strain of rats and the same feed—to avoid any confounders, yet the result should not depend on *“being done at different places, at different times, by different people”.* To finally prove that low-dose NMDR are a solid fact, such a multi-laboratory study evaluating a well-defined, toxicologically relevant apical endpoint defined a priori would be essential. It does matter*,* for example, whether the effect of the extremely low dose of 0.25 μg/kg bw/d of BPA on the mammary gland of offspring reported by Vandenberg et al. (Vandenberg et al. 2008) or the effects of 0.5 μg/kg bw/d of di-ethylhexyl-phthalate reported by Do et al*.* on testosterone formation in the offspring (Do et al. 2012) are “*just an experiment”* or reproducible scientific facts. These are results at doses around 1000–100,000-fold lower than those used in classical toxicological tests on consumer chemicals—thus we need reproducibility data to know whether, based on these observations, toxicological testing should be redefined. Given the unorthodox view on reproducibility cited above (Vandenberg et al. 2012), it appears not surprising that such an attempt at independent, multi-laboratory replication is still lacking.

In an attempt to clarify this situation, the CLARITY-BPA studies were initiated by the National Toxicology program in the Unites States. BPA was tested both at high and low doses in parallel to ethinyl estradiol (EE_2_), first in a developmental study from gestation day 6 through postnatal day 90 (Delclos et al. 2014) and then in a 2-years chronic study (Camacho et al. 2019). In both studies, a wide range of standard toxicological endpoints was analyzed. In the second study, parallel, blinded samples were supplied to specific academic laboratories working in the ED field to test for additional, non-conventional endpoints which are not part of guideline studies. These studies are too large to be reviewed here in detail, and controversial discussions on their interpretation are still ongoing. However, regarding the subject of reproducibility of low-dose NMDR effects, two comments on CLARITY-BPA can be made:

(i) Both the 90 days study and the core result generated by the NTP for the chronic study found no evidence for consistent low-dose NMDR effects on estrogen sensitive endpoints, while the positive control EE_2_ yielded clear effects on estrogen sensitive endpoints (Badding et al. 2019; Camacho et al. 2019; Delclos et al. 2014). In the 90-days study, the high doses of BPA of 100,000 and 300,000 µg/kg bw/d clearly showed adverse effects which are consistent with the weak estrogenic MoA of BPA. Especially female histopathology and estrous cycling parameters in the 300,000 µg/kg bw/d BPA and the 0.5 µg/kg bw/d EE_2_ dose groups were similarly affected (Delclos et al. 2014), showing the high potency difference between the two test substances (> 5 orders of magnitude) in vivo, while the potency difference is 7.2 × 10^4^-fold in vitro (OECD 2016). A similar in vivo potency difference and a lack of low dose effects for BPA was also shown in the uterotrophic assay and with in vivo gene expression data (Punt et al. 2013; Naciff et al. 2005; Ashby and Odum 2004). In the core data of the chronic study no evidence of low-dose effects was found, the only adverse effect observed was a *possible* relationship between the increased incidences of lesions in the female reproductive tract and the male pituitary and the highest BPA dose (25,000 µg/kg). Thus the analysis by the NTP could not confirm the low-dose NMDR hypothesis for BPA even with the most thorough repeated-dose and chronic studies ever done on BPA and analyzing a comprehensive range of classical toxicological endpoints.
(ii) The laboratories testing non-guideline endpoints found mixed results, some claiming evidence of low-dose NMDR (Heindel et al. 2020). The claimed NMDR occurred at different doses, depending on the endpoint, and had W-, Z- or U-forms—in most cases individual doses anywhere in the dose range showed a signal which was considered biologically relevant by the authors, although from a traditional statistical viewpoint, these curves would rather be interpreted as spurious or erratic. What is striking in that analysis is that the effects are in most cases not systematically compared to previous findings: was an effect on exactly the same apical endpoint observed, in the same direction, with the same statistical assessment at similar doses and of similar magnitude as observed in earlier studies? This question is more important than to ask: do we find *anything*, on *any endpoint parameter,* at *any dose* in *any direction* with *any statistical* test. (See also below about the EFSA opinion on NMDR for CLARITY-BPA). Some high-level comparison to previous studies was given in Table 5 in the recent review on the studies investigating non-guideline endpoints (Heindel et al. 2020), however this Table indicated whether effects at any dose on an organ were seen, not whether the same dose triggered the same effect on the same measurement parameter as observed before. Although the CLARITY-BPA study was conducted blinded, some of these assessments are post hoc analyses after un-blinding. See Appendix A for a detailed discussion on the study on the mammary gland development (Montevil et al. 2020) highlighted as the study providing “the strongest evidence of non-monotonicity within the CLARITY study” by Soto et al. (Soto et al. 2021)*.* What is claimed as strongest evidence might actually be interpreted as a very selective presentation of data in a post hoc evaluation (Appendix A).

Based on the above analysis, it appears that the first scientific principle, namely that reproducibility of experiments underlying a paradigm-shifting hypothesis needs to be demonstrated, has not yet been met for the low-dose NMDR hypothesis, neither by a multi-laboratory study with blinded dosing samples tested according to a standard protocol, nor by the CLARITY-BPA study.

## NMDR effects for BPA?—the view of EFSA

While I am not aware of a published, consolidated view of ECHA on the low-dose NMDR hypothesis for chemicals such as BPA, EFSA recently published a draft assessment on NMDR (EFSA 2021). This assessment included an assessment of the evidence for NMDR for BPA compiling data from an earlier report (Beausoleil C 2016), but also newer literature references including the CLARITY-BPA studies on non-guideline endpoints. EFSA discussed the different studies regarding their statistical indications for NMDR, their biological plausibility and consistency between studies for a given endpoint. The report concluded for BPA that: “Statistical assessments have identified some NMDR datasets extracted from the Clarity study, e.g. for weight at specific time points. However, for each outcome there is a lack of consistency across existing studies.” For the CLARIY-BPA study by Montevil (Montevil et al. 2020) recently highlighted (Soto et al. 2021) as a key effect observed in CLARITY and discussed here in Appendix A, EFSA concluded “In the absence of any clear biological explanation why the dose response curve may behave in such non-linear manner and taking into consideration lack of overall significance (from the NULL model) the pattern observed may be a result of overfitting of the data rather than a true biological relationship. In any case, the findings from this paper need to be replicated before any conclusions on relevance and adversity can be made.”

Thus, EFSA currently does not support that low-dose NMDR are of relevance for BPA risk assessment,^1^ and does not regard the results on non-guideline endpoints from the CLARITY-BPA consortium as consistent findings. This indicates that neither the US authorities nor EFSA came to a conclusion supporting the low-dose NMDR hypothesis for BPA. Since BPA is the starting point for the whole hypothesis for low-dose NMDR, it needs to be questioned whether the no-threshold approach for ED based on the NMDR hypothesis is valid and should be the basis for new regulation.

## In vitro ED testing proposed under REACH—implementing not fully validated tests as regulatory requirements

ECHA recently proposed to implement a battery of ≥ 7 in vitro endpoints for the assessment of all new chemicals (REACH Annex VII) (Commission 2021b). Some of the in vitro assays are covered in OECD guidelines (OECD 2016, 2020). It is often stated that these are *validated* and *performance-based* assays, but to many stakeholders it is not clear what *validated* means in this case: validation studies are conducted to assess reliability (i.e., the extent of intra- and inter-laboratory reproducibility) and relevance (i.e., the ability of the test method to predict or measure the biological effect of interest in an organism) (Hartung et al. 2004). The estrogen and androgen reporter gene assays covered under OECD 455 and 458 (OECD 2016, 2020) were indeed tested in detail for their intra- and inter-laboratory reproducibility with very favorable results. However, these are only two modules within a full test validation (Hartung et al. 2004). The aspect of *relevance and predictivity* to the in vivo situation is of key importance and has been assessed in detail in the validation of other in vitro OECD tests such as the ones introduced for skin irritation and corrosion, eye irritation and skin sensitization (ECVAM 2014; Spielmann et al. 2007). The resulting test guidelines thus contain a ‘prediction model’ which separates chemicals into classes, based on the prediction of an apical endpoint. On the other hand, the OECD validation of the reporter gene assays for endocrine activity calculated sensitivity, specificity and accuracy values for these in vitro tests when compared to other in vitro tests and relevance was not assessed. This is reviewed in detail in Appendix B in supporting Information for the evaluation of predictivity in the validation reports for the three assays in TG 455 as one example.

The other term, next to “*validated”,* which is also used with different meanings in an OECD context is “*performance-based*”. Thus TG 455 is called a performance-based test guideline. The same term was recently used when validating defined approaches (DA) for skin sensitization (OECD 2021). In the case of the DA project, performance-based indeed refers to the evaluation of the DA vs. animal and human reference data. For TG 455, performance-based again refers only to the performance with which a new in vitro assay can predict the in vitro results in other in vitro assays. Of course here the cat bites its own tail. The fact that the term “performance-based” at OECD is used for predictivity at completely different levels can instill a false trust that “*validated, performance-based”* tests in TG455 can now be used in a regulatory setting.

Most importantly, TG455 and 458 give decision thresholds to rate chemicals “positive”, and depending on the assays this threshold is at only 10–20% efficacy at a test concentration up to 1000 μM. These thresholds are arbitrary and are not based on any assessment vs. in vivo data and they do not come from any published scientific assessment. The low efficacy thresholds for positivity and the high required maximal test dose lead to a very high positivity rate as shown in Appendix B and summarized in Table 1: 29% of all chemicals (from a collection of 8311 chemicals) were rated as either estrogen or androgen agonists or antagonists in Tox21 screening. Furthermore, Tox21 screening routinely tested chemicals at a 12 times lower maximal concentration as compared to the maximal concentration in TG455 and TG 458. These higher test doses will lead to more chemicals passing the threshold of 10–20% efficacy, and thus the rate of 29% positives is still an underestimation of the positivity rate when applying the test guideline criteria. Finally, these are only 2 tests (four endpoints) of the test battery proposed by ECHA—by adding more tests in the proposed test battery, this overall positivity rate of chemicals having any in vitro endocrine activity will further increase. Thus, before such *in* vitro testing requirements are introduced in a regulatory setting, the tests should be fully validated, and that would include validation for relevance. Otherwise, there is a high risk that all these doubtful positive results will have to be followed up by animal tests, which would trigger thousands of unnecessary additional animal tests.

**Table 1:** Summary of the positivity rate^1^  of the Tox21 tests most closely related to tests in OECD TG 455 and 458^2^

	Active (*n*)	Active (%)	Active/inconclusive (*n*)	Active/inconclusive (%)
ER luc BG 1 agonist	937	11.3	1448	17.4
ER luc BG 1 antagonist	738	8.9	1048	12.6
ER luc BG 1 agonist and/or antagonist	1623	19.5	2375	28.6
ar-bla-agonist	426	5.1	647	7.8
ar-bla-antagonist	1383	16.6	1713	20.6
ar-bla agonist and/or antagonist	1580	19.0	1961	23.6
ER luc BG 1 agonist/ar-bla agonist combined	1206	14.5	2271	27.3
ER luc BG 1/ar-bla agonist and antagonist combined	2380	28.6	2877	34.6

^1^For details of the analysis see supporting information Annex B

^2^Inconclusives were only counted if they have an efficacy of at least 20% or inhibition of 30% for antagonists in accordance with OECD TG data interpretation criteria

## A call for reason unheard—dietary phytoestrogens as potential benchmark to bring dose–response back into the field

A call for a scientific assessment of chemicals for their estrogenic endocrine effects which starts with a potency comparison vs. widely distributed natural phytoestrogen has recently been made by eminent toxicologists and editors of the main toxicological journals. This proposal has been published simultaneously in this journal and in nine peer reviewed international Journals of Toxicology (Autrup et al. 2020). Based on potency considerations, the authors argue that most synthetic ED have lower potency than natural endocrine active substances such as phytoestrogens which are contained in our daily diet. They then proposed to use in vitro potency data not to label chemicals as ‘positive’ as done when using OECD 455 and 458 and applying a generic approach, but rather to use these potency data to decide whether higher tier studies are needed: “Regarding testing for potential endocrine effects, a scientifically justified screen should use in vitro tests to compare potencies of synthetic ED with those of reference natural ED. When the potency of the synthetic ED is similar or smaller than that of the natural ED, further testing in laboratory animals and regulatory consequences are not warranted”. With this science based approach considering potency, the majority of additional animal testing triggered by the new regulatory proposals could be avoided. There is a risk though, that this call goes unheard and potency information is completely ignored (as exemplified by the recent BP assessment) in a new system for chemical assessment and registration, which does not consider potency in the assessment, aiming at a generic approach influenced by the low-dose NMDR hypothesis.

## Outlook

The clash between traditional toxicology and the low-dose NMDR hypothesis in evaluating BPA specifically and ED in general has been called ‘the great divide’ by ED researchers over 10 years ago (Vandenberg et al. 2009). Indeed, as illustrated here by the independent, recent assessment of the two parabens, this ‘great divide’ on how to assess ED now goes straight through the European regulatory bodies. Obviously this divide now also includes the thinking behind basic scientific principles such as reproducibility, potency and biological plausibility. In a recent commentary (McIlroy-Young et al. 2021) on this divide between risk and hazard-based assessments on ED and between classical toxicologists and ED researchers, it was stated that *“it is time to retire consensus thinking in regulatory toxicology and make way for methods, processes, and tools that embrace a plurality of viewpoints”* stating “*there is some truth to both stories”* (McIlroy-Young et al. 2021)*.* However, as illustrated here, the gap on the lowest doses showing adverse effects for BPA between these opposing views is around five to six orders of magnitude wide. Thus, assuming that either side of this great divide and wide potency gap ‘has some truth’ cannot be correct. Science is not about compromise—science is based on the assumption that we start with objective, reproducible and testable facts. Judgments on how to derive thresholds, margins of safety and acceptable risks and hence regulatory consequences are then indeed subject to value judgments and need deliberations. But first we need to know: is it true that chemicals such as BPA and phthalates have reproducible effects on the physiological system at doses of ≤ 1 μg /kg bw/d, i.e. far over 1000-fold below the range where toxicological assessments of consumer chemicals at ECHA normally operate. Only if this scientific question is answered we can assess whether the no-threshold assumption for ED and their generic classification as SVHC is scientifically founded and define which regulatory tools are needed.

From other scientific fields we do have all the necessary tools to answer the outstanding questions highlighted in this commentary: the field of alternatives to animal testing has progressed and has shown how relevance vs. the in vivo outcome can be assessed. Equally, that field has shown the power of blind-coded inter-laboratory trials to prove reproducibility. This is also similar to the general practice of double-blinded randomized clinical studies to approve new drugs. If these tools are properly applied, the low-dose NMDR hypothesis can ultimately be tested, in vitro methods can be validated for relevance and regulation built accordingly.

## Supplementary Information

Below is the link to the electronic supplementary material.

Supplementary file1 (DOCX 39 KB)

## Footnotes

1 Note: EFSA also evaluated NMDR for phthalates. From a plethora of studies, they identified the study of Do et al. (2012) as the key study to support NMDR for di-ethylhexyl-phthalate with several other studies giving supporting evidence, indicating that in the case of di-ethylhexyl-phthalate, NMDR are plausible. However, the Do et al*.* study found effects at the extremely low concentration of 0.5 µg/kg bw/d and had some 100-fold spacing factors in the dose–response measured. Also the study accepted a *p* < 0.1 for significance level for fetal serum testosterone levels. The supporting evidence identified by EFSA comes from studies conducted at 10′000 µg/kg bw/d, i.e. a completely different dose. Thus, also for the Do et al*.* study, one may ask for reproducibility of the effects noted at 0.5—1 µg/kg to draw firm conclusions.
