# Supplementary material for: Scientific discrepancies in European regulatory proposals on endocrine disruptors—REACH regulation quo vadis?
Source: Arch Toxicol. 2021 Sep 10;95(11):3601–9. doi: 10.1007/s00204-021-03152-7 (PMC8492591; doi:10.1007/s00204-021-03152-7)
Supplement: Supplementary file 1 — Supplementary file1 (DOCX 39 KB) [file 204_2021_3152_MOESM1_ESM.docx]

**Supporting information to “Scientific discrepancies in European regulatory proposals on endocrine disruptors – REACH regulation *quo vadis*?”**

**Appendix A. Proof for low-dose NMDR? -The academic CLARITY-BPA study by Montevil et al.**

In a recent review in *Nature Endocrinology*, Soto *et al.* (Soto et al. 2021) reviewed their own CLARITY-BPA study (Montevil et al. 2020), claiming that: “**This study provided the strongest evidence of non-monotonicity within the CLARITY study, thus validating the theoretical stance in endocrinology regarding the prevalence of NMDRCs**”. Thus it is interesting to scrutinize this key study for the claim that studies by academic labs working on non-guideline endpoints proved the NMDR hypothesis within the CLARITY consortium, differently from the core studies evaluated by the National Toxicology Program.

No Figures are reproduced here from the study, but the study is freely available under: <https://ehp.niehs.nih.gov/doi/10.1289/EHP6301>

Montevil *et al.* (Montevil et al. 2020) showed in Figure 4 overall effects on scores for mammary gland development. Ethinyl estradiol at 0.5 μg/kg (EE2) had statistically significant effects on accelerated gland development, while no effect was noted on gland development neither for EE2 at 0.05 μg/kg nor for any dose of BPA up to 25’000 μg/kg, indicating that, on this estrogen sensitive endpoint, the *in vivo* potency difference between EE2 and BPA was > 50’000 fold. This is consistent with the > 10^5^ fold *in vivo* potency difference observed in the 90 days CLARITY sub-chronic study (see main text) and the *in vivo* potency difference in the uterotrophic assay of 2 × 10^6^ - fold between EE2 and BPA (Punt et al. 2013). Next to the overall score of gland development, Montevil *et al.* performed a very detailed morphometric analysis of the mammary glands and assessed in total 92 morphological parameters. They performed unsupervised principal component analysis (PCA) on these multivariate data, and found that the first two PCA axes explained 60% of the variation in the dataset and did completely separate the 0.5 μg/kg EE2 samples from the control, all the BPA doses and from the EE2 at 0.05 μg/kg treated animals, which clustered together, confirming the observations on the overall gland development scores, that none of the BPA doses between the 2.5 and 25’000 μg/kg dose had any comparable *in vivo* estrogenic effect to 0.5 μg/kg EE2.

**Thus, these two independent analyses of gland development are very consistent and convincing, proving the *in vivo* estrogenic effect of EE2 at 0.5 μg/kg and lack of such an effect of BPA up to the top dose** (which in the chronic study is below the one reported for typical estrogenic effects *in vivo* of BPA)*.* Interestingly, in their Nature Endocrinology review (Soto et al. 2021) of their own data, Soto *et al.* do not at all mention this analyses of the overall data of their own study looking at the entire dataset and the comparison of BPA to EE2, which actually gives a very clear picture that BPA up to the tested dose *did NOT* act like the estrogen EE2 supporting this tremendous potency difference.

Montevil *et al.* further analysed the data and claimed that starting at the third dimension in the PCA plot, differences are seen between doses (Figure 5B in their analysis), however the third dimension explains only 7.9% of the overall variation in the data and it still does not separate the groups. Only when the positive control was omitted from the dataset (Figure S5D), the 25 μg/kg BPA and the 250 μg/kg treatment became just about separated in the plot of the second and third PCA axes only (and still not in the two first dimensions explaining most of the variability), but they were apparently not separated from the control treatment. However – it is difficult to judge any other comparison in the PCA plot shown, as only median and not individual values are plotted, and circles representing the variability are selectively shown only for 25 μg/kg and the 250 μg/kg BPA treatments, but omitted for other doses and the control. This is a highly unconventional way of selectively depicting the data variance in PCA as it only indicates variability of selected groups and hence hides the true variation of the dataset.

The study then went into detailed analysis to test whether there is anywhere in the dataset a change between two consecutive doses, reporting a ‘breaking point’ between the 25 and the 250 μg/kg dose. For single endpoints out of the 92 endpoints, they found a difference between these two doses, although variation is high (Figure 8 in their paper). They draw a Z-shaped dose-response into their data, which is claimed to prove the non-monotonic effect, and they claim the same effect is seen for multiple endpoints – however, even for the 6 selected endpoints out 92 shown, this curve shape is seen for three endpoints, while the break occurs at a higher dose for one, is very spurious for another and there is a W-shape in another endpoint (despite the fact that a Z-shape is manually drawn into the data), with no data for 86 more endpoints shown. The authors go on to claim that the same effect was seen in independent cohorts of animals confirming these observations. However, what they show in Figure 9 to prove this claim on same effect in different cohorts **are actually two different endpoints selected** from the 92 endpoints than in Figure 8 and even for those the effect of Z-shape is spurious. In any case: Claiming that an observation is confirmed by selecting any other endpoint form a multi-variate dataset with 92 variables and comparing curve shape of those different variables tested in different study cohorts appears to violate basic scientific principles.

What is most important in this study: The idea of the (academic) CLARITY-BPA studies was to find whether, by blinded analysis, low dose NMDR effects reported earlier can be reproduced. While this scientific approach is nicely fulfilled by the unsupervised PCA analysis summarized above (which did not find BPA effects, and is now dismissed in the discussion by Soto et al. (Soto et al. 2021)), what Montevil present in the second part of the analysis does not yield a proof for reproducibility of low-dose NMDR of ED by the so much needed blinded analysis for multiple reasons:

- It is a *post hoc* analysis which could only be done after un-blinding when it was known which samples came from consecutive doses.
- It checks whether there is anywhere a change between consecutive dose-groups, in any direction and on any of the 92 parameters. Such a statistical *post hoc* analysis in a multivariate dataset (92 parameters × 5 doses × 2 directions = 920 comparisons between consecutive doses and control) can easily find some patterns – but as the PCA analysis shows, only a very minor fraction of overall data variability is between the BPA groups and the control, and PCA does not separate groups.
- The claimed Z-shaped NMDR was not found for an endpoint tested in the same way before, to check for reproducibility of a prior finding. (It indeed did test mammary gland development tested multiple times before, but not on the same endpoint / measurement parameter.)
- There is no discussion whether the same NMDR, with the same shape was observed before on the same endpoints shown in Figure 8 and whether the breaking point was observed between the same doses before.
- Claim for reproducibility are made between different CLARITY cohorts of animals – but different parameters are evaluated in different cohorts to substantiate this claim.

Thus, a global analysis of the Montevil study indicates, that if the claim by Soto *et al.,* that “**This study provided the strongest evidence** of non-monotonicity within the CLARITY study” is correct, then it appears that evidence for NMDR from CLARITY-BPA is indeed very weak and can only be made by selectively picking data from a large dataset, selectively depicting only part of the variability on PCA plots, looking at different endpoints in different co-horts, omitting the positive control form analysis and drawing NMDR of any shape into a variable dataset with high standard errors. These seem to me unconventional ways of working with multivariate data.

**Appendix B. Validation status and positivity rate of the *in vitro* tests for (anti)estrogenic and (anti)androgenic mode of action in OECD TG 455 and 458 – detailed assessment**

1. **Validation status: Assessment of the predictivity / relevance module in test validation**

The predictivity module in comparison to *in vivo* data was not a key part of the OECD validation of the reporter gene assays for endocrine activity in TG 455 and 458. Sensitivity, specificity and accuracy values were calculated for these *in vitro* tests **when compared to other in vitro tests**. Below is a summary of the evaluation for predictivity in the validation reports for the three assays in TG 455 as one example.

- For the BG1Luc Estrogen Receptor, the ICCVAM validation study (ICCVAM 2011) assessed 42 recommended substances vs. an ICCVAM consensus call. Among these, 35 could be assessed based on the experimental data obtained. However, these 42 chemicals reference calls are mainly **based on *in vitro* data and not from an evaluation of *in vivo* data**. In addition, the validation report contains data from the uterotropic assay on 13 chemicals among these are only 2 negatives (one being correct-negative and one false-positive in the *in vitro* assay*s,* see Tables 5-14 and 5-15 in the validation report, (ICCVAM 2011)). Therefore the BG1Luc Estrogen Receptor assay cannot be considered as a test validated for predictivity of *in vivo* data based on the validation report.
  In addition, a minimal efficacy of 20% vs. the efficacy of estrogen at a non-cytotoxic concentration not leading to limited solubility up to a maximal test concentration of 1000 μM leads to a positive call. There is actually no background literature indicating that such a weak partial agonistic activity at such high concentration has any *in vivo* relevance. Thus these decision thresholds in the data interpretation procedures had never been scientifically scrutinized and it is not clear how it was derived.
- For the ERα CALUX assay, the validation study only compared classification against *in vitro* data from the other validated *in vitro* assays. However the validation report cites an *in vitro* to *in vivo* correlation analysis, which tested 30 synthetic estrogens and other hormone derivatives both *in vitro* and *in vivo* (Sonneveld et al. 2006). This reference indeed reports a very elegant and comprehensive study to estimate potency prediction of synthetic hormone analogues by the *in vitro* method. However, it did not include typical industrial chemicals the assay is intended to be used for, nor did it test classification accuracy based on the data interpretation criteria set down in TG 455. Therefore also ERα CALUX assay, with the decision threshold of TG 455, cannot be considered as a test validated for predictivity of *in vivo* data.
- Finally, the predictivity for uterotrophic data was best studied in the validation report on the hER-HeLa-9903 cell line assay (OECD 2015), which included an *in vitro* to *in vivo* correlation for 48 chemicals with uterotropic assay data, among them 16 *in vivo* negatives. This analysis yielded a high predicitivity (91% sensitivity and 88% specificity). However this result was only obtained if chemicals were rated positive in case of at least 50% receptor activation as compared to E2 at a maximal test concentration of 10 μM (Table 16 in the validation report). The final data interpretation criteria included in the test guideline, though, is based on a threshold of 10% activation: With this criterion for efficacy (but still with a maximal test concentration of only 10 μM), the specificity of the assay drops to 50% (calculated from Table 16 in the validation report). Furthermore, as indicated, the validation of the hER-HeLa-9903 assay included a top concentration of 10^-5^ M (The report stated: “*On the basis of sensitivity of the assay system, the concentration range at 10^-11^ -10^-5^M can detect estrogenic activity of well-known weak estrogenic chemicals*”), however the OECD TG now includes a top concentration of 1000 μM for this assay (unless chemicals are insoluble or cytotoxic at this concentration), i.e. a 100-fold higher concentration than in the test validation. The specificity vs. the uterotrophic assays (which is already at 50% by lowering the threshold to 10% efficacy) will certainly further drop dramatically by increasing the test dose 100-fold. To my knowledge this was not studied nor discussed in any public document and it is unclear why these modifications of the data interpretation criteria of the assay were introduced into the test guideline, other than to predict the outcome of the other *in vitro* assays. It appears that there are no scientific data to justify these changes and no scientific indication that a chemical with 10% efficacy at 1000 μM has any *in vivo* activity. Therefore, also for the hER-HeLa-9903 with the data interpretation procedures implemented in OECD 455, there is no validation for predictivity.

This lack of validation for predictivity was obviously known at the time of writing the test guideline: The OECD guideline thus did not define these thresholds to rate chemicals as positive with the classical term ‘prediction model’, but used the term “data interpretation criteria”. The OECD guidelines 455 and 458 clearly state that “*the outcome of the tests* ***cannot be used on their own for safety assessment decisions and only be used for screening and prioritisation purposes***”.

Here we only reviewed the status for the estrogen agonist assays, which is the poster child of *in vitro* endocrine assays. It is even less clear what the *in vivo* relevance is for the outcome of the antagonist assays conducted under the conditions laid down in TG 455 and TG458, but conducting and reporting these assays is part of the guideline and would become mandatory with the proposed data information requirements.

**b) Positivity rate of *in vitro* testing: The Tox21 database as a large case-study**

What is clear from looking at the low efficacy thresholds for positivity and the high required maximal test concentration (which would only be limited by 1000 μM maximal concentration or cytotoxicity / insolubility) is that a high number of false-positive screening results might be generated, if the OECD tests with their current data interpretation procedures will be implemented as Information Requirements in Annex VII. Currently, we are not aware of reports on the positivity rate of screening random chemicals with the thresholds set in TG455 and 458. However, we can investigate the positivity rate in the Tox21 screening which used one of the cell lines of TG455 and which screened ER and AR agonist/antagonists for 8311 chemicals. It is important to note, the Tox21 screening routinely **tested chemicals up to 80 μM, i.e. at a 12 times lower concentration as compared to the maximal concentration in TG455 and TG 458**^[[1]](#footnote-1)^.

**Analysis of results from the ER luc BG1 estrogen assay**

The Tox 21 screening includes two tests for estrogen receptor agonism/antagonism. As the ER luc BG1 agonist/antagonist assay is based on the same cell line used in TG 455 and hence equivalent, here the results of this assay are evaluated.

Out of 8306 different CAS numbers tested, 937 (11.2%) chemicals are labelled active agonists in the Tox21 ER screening, while 738 (8.9%) are active antagonists. In total 1623 chemicals (19.5%) are either agonist or antagonists. It is not known, how far this value increases by raising the test concentration 12-fold as required in TG455 and for how many chemicals this is possible based on solubility and cytotoxicity, but in general as the test concentration increases, unspecific reactions will certainly increase.

When including the chemicals which are considered inconclusive agonists or inconclusive antagonists, the frequency of positives raises to 17.4% for agonists, 12.6% for antagonists and 28.6% for combined agonist/antagonists. Chemicals are rated inconclusive due to issues with the curve shape or reproducibility, but all chemicals with an “inconclusive agonist” or “inconclusive antagonist” call counted here have either 20% efficacy in agonist or 30% inhibition in antagonist mode, i.e. at least fulfil this decision thresholds of the guidelines, albeit tested only at lower maximal concentration. It is not clear, how many of these would be rated as positive when applying the criteria in TG455 for curve evaluation, reproducibility and maximal test concentration, but a significant fraction certainly would.

**Analysis of the results of the Tox21-ar-bla assay**

The Tox 21 screening includes two tests for androgen receptor agonism/antagonism. The AR mda kbluc assay technically is most closely related to the tests in the TG458 (a classical nuclear receptor reporter gene assay). However, it contains the MMTV promoter containing response elements for both androgen receptor (AR) and glucocorticoid receptor (GR), and may thus be less specific. Some assays in TG458 specifically were designed to limit glucocorticoid response cross-talk. Thus we here analysed the data for the more specific Tox21-ar-bla assay, which has a lower positivity rate.

Out of 8306 different CAS numbers tested, 426 (5.1%) chemicals are labelled active agonists in the AR screening, while 1383 (16.5%) are active antagonists, in total 1580 chemicals (19.0%) are either agonist or antagonists. It is not known, how far this value increases by raising the test concentration 12-fold as required in TG458.

When including the chemicals which are considered inconclusive agonists or inconclusive antagonists, the frequency of positives raises to 7.8% for agonists, 20.6% for antagonists and 23.6% for combined agonist/antagonists. Chemicals are rated inconclusive due to issues with the curve shape or reproducibility, but all chemicals with an “inconclusive agonist” or “inconclusive antagonist” call counted here have either 20% efficacy in agonist or 30% inhibition in antagonist mode. It is not clear, how many of these would be rated as positive when applying the criteria in TG458 for curve evaluation, maximal test concentration and reproducibility.

**Impact on positivity rate by combining the results of the multiple assays**

The proposed information requirements for Annex VII include multiple tests. Thus if we combine all calls for agonists from either the ER or the AR assay, 1206 chemicals (14.5%) are agonists, while 2380 chemicals (28.6%) are labelled as either agonist or antagonist in one of the two assays. This value is raised to 34.6% of chemicals including the inconclusive agonists/antagonist calls. These values on overall fraction of chemicals with a positive rating in any *in vitro* ED screening assay of the proposed test battery almost certainly will further increase because:

1. the required test concentrations are higher in TG455 and TG458 as compared to the Tox 21 screening and
2. since the proposed information requirements ask for further testing on H295R steroidogenesis assay, aromatase inhibition and one or multiple yet to be defined thyroid assays. This extended battery will certainly further raise the overall positivity rate.

Table 1. Summary of the analysis of the Tox21 tests most closely related to tests in OECD TG 455 and 458

|  | Active (n) | Active (%) | Active/ inconclusive (n) | Active/ inconclusive (%) |
| --- | --- | --- | --- | --- |
| ER luc BG 1 agonist | 937 | 11.3% | 1448 | 17.4% |
| ER luc BG 1 antagonist | 738 | 8.9% | 1048 | 12.6% |
| ER luc BG 1 agonist and/or antagonist | 1623 | 19.5% | 2375 | 28.6% |
| ar-bla-agonist | 426 | 5.1% | 647 | 7.8% |
| ar-bla-antagonist | 1383 | 16.6% | 1713 | 20.6% |
| ar-bla agonist and/or antagonist | 1580 | 19.0% | 1961 | 23.6% |
| ER luc BG 1 agonist / ar-bla agonist combined | 1206 | 14.5% | 2271 | 27.3% |
| ER luc BG 1 / ar-bla agonist and antagonist combined | 2380 | 28.6% | 2877 | 34.6% |

^1)^ inconclusives were only counted if they have an efficacy of at least 20% or inhibition of 30% for antagonists in accordance with OECD TG data interpretation criteria

**c) Screening for prioritisation?**

The counter-argument to above raised concerns on (i) lack of validation for predictivity, the (ii) lack of scientific validity of the data interpretation procedures /thresholds selected and (iii) the issue of very high positivity rate from the proposed test battery could be that these tests are intended “only for screening and ***prioritisation purposes”,*** which actually is the sole purpose of these tests as stated in the OECD guidelines. However, this argument is only valid if:

- These tests are used in the same REACH Annex, in which higher tier studies would also be mandatory to follow up on potential screening results. In such a case, a negative screening result could indeed be used for a waiver of some higher tier studies, even if the (false)-positivity rate is high.
- In addition, a fixed waiver for higher tier studies **in case of negative *in vitro* results would then be needed to be implemented within the corresponding Annexes.** Else, the assays do not fulfil the prioritisation purpose for which they were intended.

Based on these considerations, it does not appear that the tests, if used in REACH Annex VII, would be applied according to the purpose they are described in the OECD TG – and these assays are thus *not fit for the purpose* for which they are currently being proposed.

**Practical implications**

- Based on the data interpretation procedures of the OECD guideline, a large fraction of the chemicals, if not a majority, would be rated ‘positive’ in at least one assay from the proposed *in vitro* battery. If ECHA applies the low-dose NMDR assumption and ignores potency considerations as in the report on BP, these positive results can only be further assessed with higher tier animal studies which would lead to **a large number of animal tests** triggered by the high positivity rate.
- Companies in the **cosmetic sector** will be in a complex situation, as they should not proceed to animal testing, due to the **ban on animal** testing but then will have these screening results in their dossier without a mean to prove non-relevance.

**Background on the data analysis: Parameters and data source used to analyze the Tox21 data**

The following files form The Tox21 resources were used:

- ER agonists: tox21-er-luc-bg1-4e2-agonist-p2.aggregrated.txt
- ER antagonists: tox21-er-luc-bg1-4e2-antagonist-p2.aggregrated
- AR agonists: tox21-ar-bla-agonist-p1.aggregrated
- AR antagonists: tox21-ar-bla-antagonist-p1.aggregrated

In each case, the column “Channel_outcome” was used. This column integrates data from multiple repetitions and in the antagonist mode also integrates the data from the cytotoxicity counter screen. This has been described in detail in the supporting information of (Huang et al. 2014), Table S3. The databases were filtered for either “active agonist” or “inactive antagonist”, and chemicals with multiple entries were calculated as positive if at least one of the entry was positive removing the duplicate call for all those with several entries (several CAS numbers have multiple entries in Tox21). For inclusion of the inconclusives, “active agonist” and “inconclusive agonist” calls were pooled, and a chemical was again counted if it had at least one of these calls. The total number of entries in Tox21 is over 10’000, but all was calculated based on unique CAS numbers, reducing the database to the 8311 chemicals cited in (Huang et al. 2014).

**References**

1. Soto, A.M., C.M. Schaeberle, and C. Sonnenschein, From Wingspread to CLARITY: a personal trajectory. Nat Rev Endocrinol, 2021. 17(4): p. 247-256.

2. Montevil, M., et al., A Combined Morphometric and Statistical Approach to Assess Nonmonotonicity in the Developing Mammary Gland of Rats in the CLARITY-BPA Study. Environ Health Perspect, 2020. 128(5): p. 57001.

3. Punt, A., et al., Effect of combining in vitro estrogenicity data with kinetic characteristics of estrogenic compounds on the in vivo predictive value. Toxicol In Vitro, 2013. 27(1): p. 44-51.

4. ICCVAM. ICCVAM Test Method Evaluation Report The LUMI-CELL® ER (BG1Luc ER TA) Test Method: An In Vitro Assay for Identifying Human Estrogen Receptor Agonist and Antagonist Activity of Chemicals. NIH Publication Number 11-7850 2011; Available from: https://ntp.niehs.nih.gov/iccvam/docs/endo_docs/erta-tmer/bg1lucer-ta-tmer-combined.pdf.

5. Sonneveld, E., et al., Comparison of In Vitro and In Vivo Screening Models for Androgenic and Estrogenic Activities. Toxicological Sciences, 2006. 89(1): p. 173-187.

6. OECD. REPORT OF THE INTER-LABORATORY VALIDATION FOR STABLY TRANSFECTED TRANSACTIVATION ASSAY TO DETECT ESTROGENIC AND ANTI-ESTROGENIC ACTIVITY. ENV/JM/MONO(2015)34 2015; Available from: https://www.oecd.org/env/ehs/testing/37504278.pdf.

7. Huang, R., et al., Profiling of the Tox21 10K compound library for agonists and antagonists of the estrogen receptor alpha signaling pathway. Sci Rep, 2014. 4: p. 5664.

1. For the CALUX assays in TG455 and 458 the maximum test concentration in absence of cytotoxicity or solubility issues is 100 μM, while for all other assays it is 1000 μM. It is unclear what the scientific rationale is behind such a difference for assays on the same endpoint. [↑](#footnote-ref-1)
